# Investigation into the Sonodynamic Activity of Three Newly Synthesized Derivatives of Ciprofloxacin

**DOI:** 10.3390/molecules29163735

**Published:** 2024-08-07

**Authors:** Ying Zheng, Jing Lv, Jun Zhang, Yu Liu, Xiaofang Wang, Bin Liu

**Affiliations:** 1 Artemisinin Research Center, China Academy of Chinese Medical Sciences, Beijing 100700, China; yzheng@icmm.ac.cn; 2 School of Pharmaceutical Sciences, Liaoning University, Shenyang 110036, China

**Keywords:** ciprofloxacin derivatives, sonodynamic damage, bovine serum albumin, *Escherichia coli*, reactive oxygen species, sonosensitizers

## Abstract

Sonosensitizers play a crucial role in the efficacy of sonodynamic antitumor therapy (SDT) and sonodynamic antimicrobial chemotherapy (SACT), highlighting the necessity for the development of new compounds with good sonodynamic activity. In this study, three novel 3-substituted ciprofloxacin derivatives (CIPD1, CIPD2, and CIPD3) were designed and synthesized. Their sonodynamic activities were evaluated by assessing the damage to bovine serum albumin (BSA) and *Escherichia coli* (*E. coli*). Furthermore, the potential mechanism underlying their sonodynamic damage activities was investigated by detecting reactive oxygen species (ROS) under ultrasound irradiation (US). The results demonstrated that all three derivatives exhibited enhanced sonodynamic damage to BSA and *E. coli* under US, with CIPD1 and CIPD2 showing superior effectiveness compared to CIP. Both the concentrations of derivatives and the duration of ultrasound irradiation were found to significantly impact their sonodynamic effects. All three CIP derivates could be activated to produce ROS following ultrasound irradiation, primarily consisting of ^1^O_2_ and ·OH. The levels of ROS production were positively correlated with their sonodynamic activities, potentially explaining the mechanism underlying their sonodynamic damage activities.

## 1. Introduction

Sonodynamic therapy (SDT) is an innovative and non-invasive tumor treatment modality that utilizes ultrasound to activate sonosensitizers, thereby inducing the generation of reactive oxygen species (ROS) for the purpose of eradicating malignant cells [1]. In comparison with alternative approaches for tumor treatment, SDT exhibits notable attributes such as exceptional focusing capability, deep tissue penetration, and minimal damage to surrounding healthy tissues [2,3]. Consequently, SDT is widely used in clinical diagnosis and therapeutic interventions and was approved by the U.S. Food and Drug Administration (FDA) for the clinical treatment of malignant tumors through the Green Channel in 2022 [4]. Following sonodynamic therapy, sonodynamic antimicrobial chemotherapy (SACT) has emerged and evolved as a promising novel approach to antimicrobial treatment. Similar to SDT, SACT also utilizes the synergistic effect of low-frequency ultrasound and a sonosensitizer for bacterial eradication. Compared to traditional antibiotic treatment, SACT has fewer adverse reactions and is prominent in the field of drug-resistant bacterial infections.

The sonosensitizers play a crucial role in the process of SDT and SACT. Under ultrasound irradiation (US), sonosensitizers transition from the ground state to the excited state. Upon returning to the ground state, they can transfer energy to the surrounding oxygen or water molecules, generating ROS [5,6]. Current research has focused on several classes of sonosensitizer-mediated sonodynamic therapies, including porphyrins and their derivatives, nanoparticles/nanoparticle complexes, antibiotics, natural product extracts, and heteranthrene dyes [7,8]. However, most sonosensitizers have poor water solubility, are less stable in the biological micro-environment, and cannot accumulate at numerous tumor sites. This makes them difficult to be widely used in clinical practice [9]. Therefore, the development of new sonosensitizers with improved sonodynamic activities is crucial for advancing the progress of SDT.

Currently, numerous researchers are dedicated to investigating the structure–activity relationship of sonosensitizers and enhancing their performance to meet the requirements of SDT. In the pursuit of new rose bengal derivatives with enhanced photodynamic and sonodynamic anticancer effects, Chen et al. [10] synthesized a series of amphiphilic rose bengal derivatives. They indicated that the introduction of a suitable methoxy polyethylene glycol fraction can enhance cellular uptake and improve intracellular ROS production, resulting in synergistic anticancer effects. In addition, Li et al. [11] developed a novel sonosensitizer by conjugating an electron donor (trianiline) and an electron acceptor (benzothiazole) to the resveratrol skeleton, which is capable of generating reactive oxygen species under low-intensity ultrasound in order to kill breast cancer cells.

The structural modification of existing compounds with sonodynamic activity is also a crucial strategy for the design and development of novel sonosensitizers. Ciprofloxacin (CIP) is a widely utilized third-generation fluoroquinolone antibacterial agent in clinical practice [12]. Our previous studies found that CIP has certain sonodynamic activities. The combination of 40 kHz ultrasound with CIP synergistically enhanced the inhibition of *E. coli* [13]. Moreover, CIP has also demonstrated remarkable antitumor efficacy against various types of tumors [14]. Its antimicrobial activity could hold significant implications for anticancer treatment as well. According to the literature, most solid tumors harbor bacteria that protect tumor cells from the immune system, promote metastasis, and are associated with chemotherapy resistance [15,16]. The efficacy of certain anticancer drugs is also attributed to their ability to target tumor-associated bacteria. For example, the chemotherapy drug 5-fluorouracil has the capability to effectively eliminate *Fusobacterium nucleatum*, a bacterium known for promoting colorectal cancer [17]. The consideration of bacterial presence within tumors is gaining traction in the field of cancer treatment. Gao et al. [18] developed a novel molecule that combines ciprofloxacin and theobromine using a new strategy of intracellular enzymatic nanofiber formation, which enabled enhanced synergistic antibacterial chemotherapy and immunotherapy. Consequently, as an antibacterial agent with antitumor effects, the structural modification of CIP has important potential in the development of novel high-performance sonosensitizers, not only for SACT, but more importantly, for SDT.

In this study, our aim was to chemically modify the C-3 carboxyl group of CIP with various substituents in order to obtain new derivatives of CIP and evaluate their sonodynamic activities in SDT and SACT. Initially, three derivatives were synthesized and structurally characterized. Subsequently, the sonodynamic activity of these derivatives was assessed through the analysis of UV–vis and fluorescence spectra, utilizing bovine serum albumin (BSA) as a model for protein damage and *Escherichia coli* (*E. coli*) as a model for bacterial inhibition. Furthermore, we investigated the impact of varying concentrations of these CIP derivatives and different durations of ultrasonic exposure on their sonodynamic activities. Additionally, we performed a comparison of the sonodynamic activities of these CIP derivatives with those of the original CIP compound. Finally, we explored the potential synergistic damage mechanism between CIP derivatives and ultrasonic irradiation by detecting ROS produced in the reaction system using an oxidation extraction spectrophotometry method. Our findings indicate that the three newly synthesized CIP derivatives exhibit significant sonodynamic activities, with CIPD1 and CIPD2 demonstrating superior efficacy and displaying greater potential as effective sonosensitizers for sonodynamic therapy.

## 2. Results and Discussion

### 2.1. UV–Vis Absorption Spectra of the Sonodynamic Effects of CIP Derivatives on BSA

**Figure 1:**
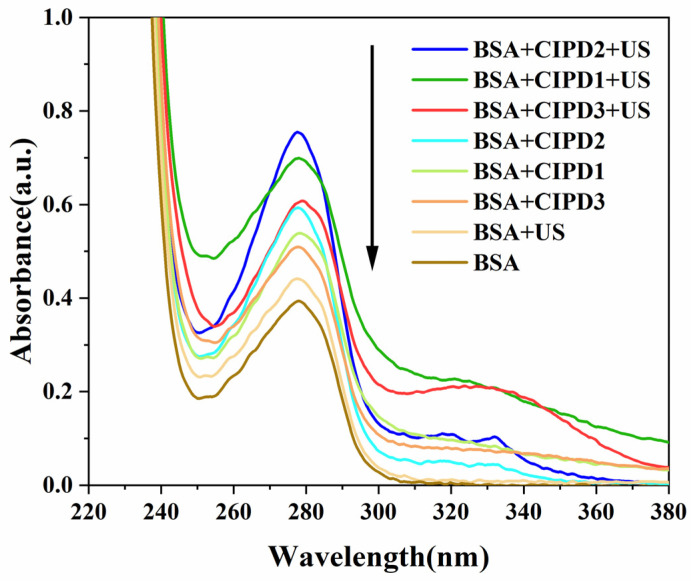
UV–vis absorption spectra of BSA in various solutions with or without derivatives and ultrasound irradiation. Ciprofloxacin derivatives: CIPD1, CIPD2, and CIPD3; US: ultrasonic irradiation. The duration of ultrasonic irradiation was 3 h. The concentration of BSA in the Tris-HCl-NaCl solution was 10 μM, and the concentrations of all three CIP derivatives in the Tris-HCl-NaCl solution were 20 μM. The black arrow indicates that the spectrum on the left corresponds to the label on the right, from top to bottom.

The absorption peak of the BSA solution was observed at 278 nm in the absence of ultrasound irradiation, as depicted in Figure 1. However, after ultrasound irradiation (BSA + US), the intensity of the BSA absorption peak increased significantly and a slight blue shift occurred. This alteration may be attributed to the disruption of the BSA’s molecular spatial structure induced by ultrasonic irradiation, leading to an increased exposure of chromogenic amino acids within the extended peptide chain.

When incorporating the CIP derivatives, the intensities of the maximum absorption peak of BSA were significantly increased in all three CIP derivative solution systems compared to that of the BSA-only solution. This phenomenon can be clearly observed both in the presence and absence of ultrasound radiation. The increased chromatic effects on BSA induced by the CIP derivatives can be attributed to the formation of new complexes between the BSA and the derivatives, resulting in conformational changes within the BSA. These interactions disrupt internal hydrogen bonds crucial for maintaining the stability of BSA’s secondary structure (α-helix), leading to the exposure of major chromophoric amino acids (such as Trp and Tyr) in BSA molecules, thereby causing a hyperchromic effect [19,20]. Therefore, it can be inferred that these three CIP derivatives may have bound to the BSA molecules and altered their spatial conformations, consequently enhancing the chromatic effects of the BSA.

Furthermore, under ultrasound irradiation, the maximum absorption peak of BSA in the CIP derivative solutions (BSA + CIPD1 + US, BSA + CIPD3 + US, and BSA + CIPD3 + US) was significantly larger than those without ultrasound irradiation (BSA + CIPD1, BSA + CIPD3, and BSA + CIPD3). These findings demonstrate the synergistic damaging effect of combining the three CIP derivatives with ultrasound on BSA.

### 2.2. Fluorescence Spectroscopy of BSA in Different Solutions

#### 2.2.1. The Effect of Various Concentrations of CIP and Its Three Derivatives

**Figure 2:**
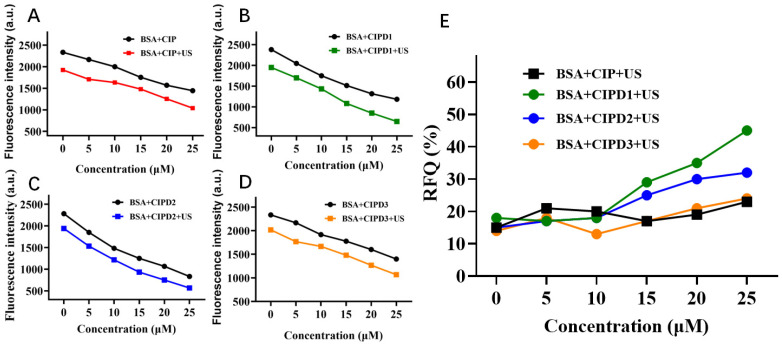
Damage effect of different concentrations of CIP and its three derivatives on BSA. Fluorescence intensities of (**A**) BSA+ different concentrations of CIP, (**B**) BSA+ different concentrations of CIPD1, (**C**) BSA+ different concentrations of CIPD2, and (**D**) BSA+ different concentrations of CIPD3 with and without ultrasound irradiation. (**E**) The relative fluorescence quenching ratios (RFQ) of CIP and its derivatives. The concentration of BSA in the Tris-HCl-NaCl solution was 10 μM. The concentrations of all three derivatives in the Tris-HCl-NaCl solution were 0–25 μM. US: ultrasound irradiation. The duration of ultrasonic irradiation was 3 h.

The impact of varying concentrations of CIP derivatives on BSA were assessed using fluorescence spectroscopy. As depicted in Figure 2A–D, the fluorescence intensities of the BSA decreased as the concentrations of CIP and its three derivatives increased. Furthermore, at any concentrations ranging from 0 to 25 μM for both CIP and its derivatives, the fluorescence intensities of the BSA in solutions decreased more significantly when subjected to ultrasound irradiation compared to those without ultrasound irradiation. This result indicates that CIP and its derivatives can be activated under ultrasound irradiation and cause additional damage to BSA molecules.

To further analyze the sonodynamic activities of different CIP derivatives, we calculated the relative fluorescence quenching ratios (RFQ) of the ultrasonically activated derivatives using the following equation:

RFQ = (F_0_ − F)/F_0_ × 100% (1)

where F_0_ represents the fluorescence intensity of the BSA + CIP derivatives solutions without ultrasound, while F represents the intensity of those solutions under ultrasound irradiation. The RFQ of the BSA with various derivatives is illustrated in Figure 2E. It can be observed from the figure that within the concentration range of 0–25 μM, the RFQ of CIPD3 remains relatively stable as its concentration increases, and its sonodynamic damage effect on the BSA is not significantly different from that of CIP. However, it is noteworthy that at concentrations greater than 10 μM, both CIPD1 and CIPD2 exhibit significantly higher RFQ values compared to CIP as the concentration increases. This indicates that the sonodynamic effect of CIPD1 and CIPD2 on BSA damage was significantly stronger than that of CIP.

#### 2.2.2. The Effect of Different Ultrasound Irradiation Durations on BSA

**Figure 3:**
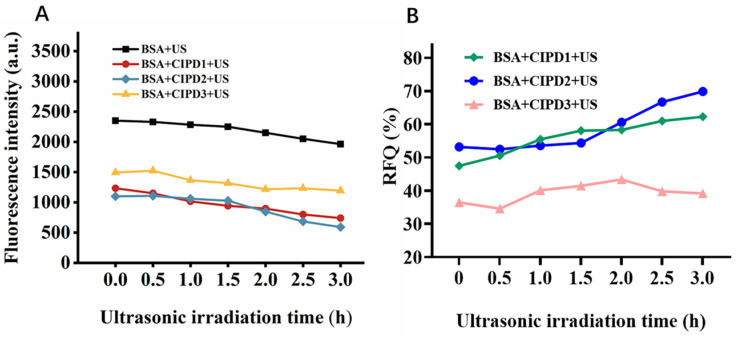
The effect of different ultrasound irradiation durations on BSA. (**A**)The fluorescence intensities (λ_ex_ = 280 nm) of BSA in various solutions under different ultrasound irradiation times. (**B**) The relative fluorescence quenching ratios (RFQ) of CIP derivatives under ultrasound irradiation. The concentration of BSA and the CIP derivatives were all 20 μM. US: ultrasound irradiation.

The fluorescence intensities of the BSA gradually decreased in all experimental groups with an increasing duration of ultrasonic exposure (Figure 3A). The introduction of the CIP derivatives led to a notable reduction in the fluorescence intensity of the BSA compared to the pure BSA solution, and it was observed that longer ultrasound exposure exacerbated the damage caused by the CIP derivatives and their synergistic effect with ultrasound on the BSA. We further calculated the RFQ of the three CIP derivatives on the BSA using formula (1). The results shown in Figure 3B indicate that the RFQ of CIPD1 and CIPD2 increased with the extension of ultrasonic irradiation time, while the RFQ of CIPD3 was lower than that of CIPD2 and CIPD1. Additionally, the RFQ of CIPD3 did not exhibit significant changes with extended ultrasonic irradiation time. The findings indicate that the combination of ultrasound irradiation with CIPD1 and CIPD2 can enhance BSA damage through a favorable synergistic effect.

### 2.3. Sonodynamic Antibacterial Activity CIP Derivatives against E. coli

#### 2.3.1. Comparison of the Sonodynamic Antibacterial Activity of CIP and Its Derivatives

**Figure 4:**
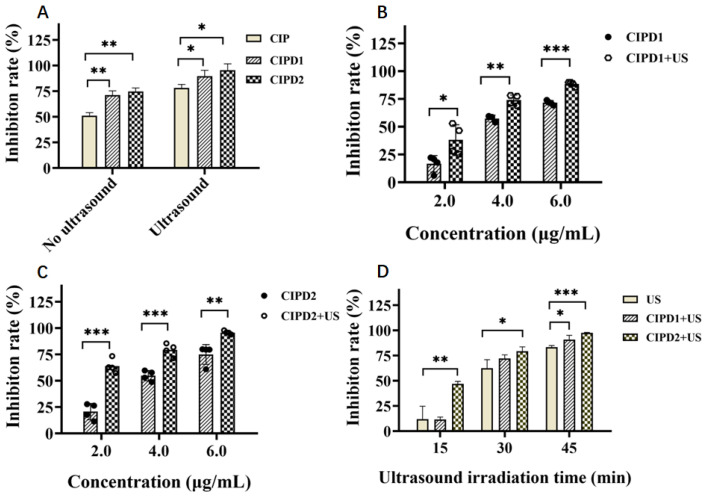
Sonodynamic antibacterial activities of CIP and its two derivatives (CIPD1 and CIPD2) against *E. coli*. (**A**) Comparison of the inhibition rates of CIP and its two derivatives at a concentration of 6.0 μg/mL with an ultrasound duration of 30 min (*n* = 3). Inhibition rates of various concentrations of (**B**) CIPD1 and (**C**) CIPD2 on the growth of *E. coli* under a 30 min ultrasound treatment (*n* = 4). (**D**) Inhibition rates of CIPD1 and CIPD2 on *E. coli* at a concentration of 4.0 μg/mL under varying durations of ultrasound irradiation (*n* = 4). The concentration of *E. coli* used in all the above experiments was 1.0 × 10^7^ cfu/mL. (* *p* < 0.05, ** *p* < 0.01, *** *p* < 0.001; US: ultrasound irradiation).

As shown in Figure 4A, even without ultrasonic irradiation, both CIPD1 and CIPD2 exhibited heightened antibacterial activities in comparison to CIP, suggesting that the structural modifications at the C-3 position of CIP improved its inhibitory effect against *E. coli*. When exposed to ultrasonic irradiation, all three compounds (CIP, CIPD1, and CIPD2) demonstrated enhanced antibacterial activities in comparison to the non-irradiated groups, suggesting that all three compounds possess sonodynamic activities. Notably, CIPD1 and CIPD2 exhibited superior sonodynamic activities compared to CIP. The enhanced sonodynamic activities may potentially be attributed to the influence of the electron donor and modifications in steric hindrance. These findings highlight that structural modifications of CIP result in increased sonodynamic antibacterial activities.

#### 2.3.2. Sonodynamic Antibacterial Activity of CIP Derivatives at Different Concentrations

Based on the observed positive sonodynamic activities of CIPD1 and CIPD2 on BSA damage, we further investigated their synergistic ultrasonic antibacterial effects against *E. coli*. As illustrated in Figure 4B,C, when combined with 30 min of ultrasonic irradiation, the antibacterial effects of CIPD1 and CIPD2 at the same concentration were significantly enhanced compared to those without ultrasonic irradiation, indicating that CIPD1 and CIPD2 have a synergistic antibacterial effect with ultrasound irradiation. Moreover, the inhibition rates of CIPD1 and CIPD2 exhibited a concentration-dependent characteristic, with their antibacterial activity increasing from concentrations of 2.0 μg/mL to 6.0 μg/mL.

#### 2.3.3. Sonodynamic Antibacterial Activities of CIP Derivatives at Different Ultrasound Irradiation Times

When the concentration of CIPD1 and CIPD2 was maintained at 4.0 μg/mL, under various ultrasonic irradiation times of 15, 30, and 45 min, CIPD1 and CIPD2 presented synergistic antibacterial effects against *E. coli* (Figure 4D). At an ultrasonic irradiation time of 45 min, the inhibitory rates of CIPD1 and CIPD2 were statistically higher than that of the ultrasound without the CIPD1 and CIPD2 group (*p* < 0.05). This result suggests that CIPD1 and CIPD2 have sonodynamic antibacterial activity and are expected to be further developed as new sonosensitizers for sonodynamic bacteriostatic therapy.

### 2.4. Exploration of the Sonodynamic Action Mechanism of CIP Derivatives

#### 2.4.1. ROS Production Induced by CIP and Its Different Derivatives under Ultrasonic Irradiation

The mechanism of sonodynamic therapy is widely accepted to be the generation of highly cytotoxic ROS through the synergistic effect of ultrasound irradiation and sonosensitizer derivatives, which can selectively target tumor cells and kill them [21,22]. To investigate the mechanisms underlying the enhanced damaging effects of CIP derivatives on BSA and *E. coli* under ultrasonic irradiation, we used an oxidation–extraction photometry method to detect the ROS generated in different mixed solution systems. Diphenyl carbazide (DPCI) was used as a ROS scavenger, which can capture ROS and generate dibenzoyl dihydrazone (DPCO) through oxidation, exhibiting a maximum absorption peak at 563 nm. The DPCO can be extracted with an organic solvent and analyzed via UV–vis spectroscopy. Its absorbance intensity at 563 nm reflects the amount of ROS generated in the solutions [23].

**Figure 5:**
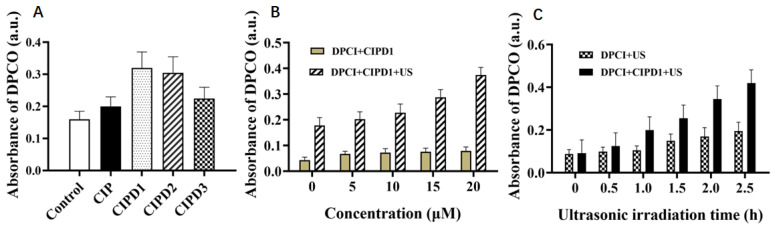
Production of ROS in different solutions detected by absorbance of DPCO (λ_max_ = 563 nm). (**A**) ROS production in CIP and its three derivatives solutions under 30 min ultrasonic irradiation. The concentrations of CIP and its derivatives were 20 μM. (**B**) Effects of different concentrations of CIPD1 on the ROS production. (**C**) Effects of varying ultrasonic times on ROS production. (US: ultrasonic irradiation).

Figure 5A shows that the addition of CIP and its three derivatives resulted in a higher absorbance of DPCO compared to the DPCI control solution, indicating that these compounds induced more ROS production under ultrasonic excitation. Additionally, both CIPD1 and CIPD2 exhibited significantly increased amounts of ROS production under ultrasonic excitation compared to CIP alone, while there was no substantial difference in ROS generation between CIP and CIPD3.

ROS are highly reactive substances containing oxygen radicals, formed through the partial reduction of O_2_ and the reaction of its products with other molecules [24,25]. In sonodynamic antibacterial therapy, ROS can target phospholipids, membrane proteins, and nucleic acids in bacterial cell membranes. This direct action results in the destruction of the cell wall and inner membrane, leading to damage to the bacterial morphological structure. Subsequently, this process causes the inactivation of the membrane transport system and the loss of protein and enzyme functions [26,27]. Therefore, it can be inferred that the sonodynamic antibacterial mechanism of CIP derivatives may be associated with the induction of ROS production under ultrasonic stimulation.

#### 2.4.2. ROS Production Induced by Different Concentrations of CIPD1 and Varying Ultrasonic Times

As shown in Figure 5B, the production of ROS increased significantly with increasing concentrations of CIPD1 ranging from 0 to 20 μM. Moreover, this increase was notably higher compared to the non-ultrasonic treatment group at equivalent concentrations, thereby demonstrating the significantly synergistic effect of CIPD1 on increasing ROS production under ultrasound excitation. As illustrated in Figure 5C, the addition of CIPD1 resulted in an increased production of ROS with the extension of ultrasound irradiation time, which was significantly higher compared to that of the DPCI-only solution. These findings highlight the importance of sonosensitizer concentration and ultrasound duration as critical factors in regulating ROS generation during sonodynamic therapy.

#### 2.4.3. Analysis of ROS in Different Solutions

The specific types of ROS produced in different solutions were further determined by employing four types of ROS scavengers. Vc is the scavenger for all kinds of ROS, while L-His is the scavenger for singlet oxygen (^1^O_2_) and ·OH. D-Man is the scavenger for ·OH, and NaN_3_ is the scavenger for ^1^O_2_. The absorbance of DPCO in the solution containing various types of ROS scavengers was compared to that without any ROS scavengers. The type and amounts of ROS production are reflected by the specific type of scavenger used and the extent of reduction in absorption values, respectively.

**Figure 6:**
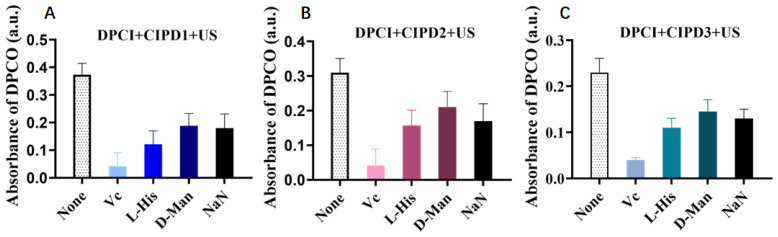
The absorbance of DPCO for (**A**) CIPD1, (**B**) CIPD2, and (**C**) CIPD3 in combination with various ROS quenchers under US. The concentrations of the CIP derivatives were 20 μM (US: ultrasonic irradiation).

From Figure 6, it is evident that the addition of each ROS scavenger led to a significant decrease in the absorbance of DPCO across all three CIP derivative solutions compared to the non-ROS scavenger group. This indicates that all three CIP derivatives can induce the generation of multiple types of ROS under ultrasound irradiation. This finding can also be inferred from the significant decrease in the quenching rate observed after adding V_C_ to each group. Specifically, the CIPD1 solution produced higher levels of ^1^O_2_ and ·OH, while CIPD2 showed higher levels of ^1^O_2_ (L-His and NaN_3_), and CIPD3 generated relatively more ·OH. Among them, CIPD1 displayed a superior capacity for ROS production.

## 3. Materials and Methods

### 3.1. Experimental Strains and Reagents

All the reagents used were of analytical grade. Bovine serum albumin (Lot. M0104A) was obtained from Beijing Biotopped Technology Co., Ltd., Beijing, China. *Escherichia coli* (CCTCC AB 93154) was kindly provided by the School of Life Sciences, Liaoning University, Shenyang, China. Ciprofloxacin Hydrochloride was purchased from Wuhan Yuancheng Co-create Technology Co., Ltd., Wuhan, China. m-Methoxyaniline, benzylamine, α-naphthylamine, and diphenylcarbazide (DPCI) were all purchased from SinoPharm Chemical Reagents Co., Ltd., Beijing, China. Beef extract and nutrient agar medium were purchased from Beijing Aoboxing Biotechnology Co., Ltd., Beijing, China.

### 3.2. Synthesis and Structural Characterization of Ciprofloxacin Derivatives

**Figure 7:**
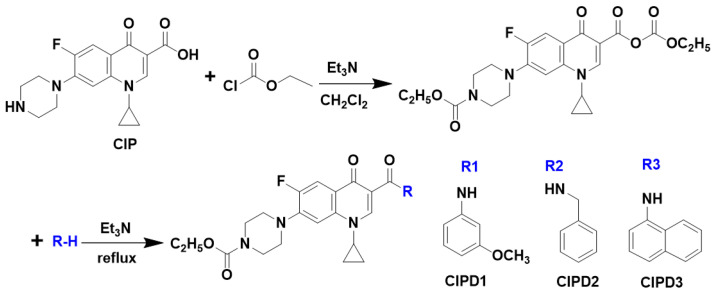
Synthetic route of the three CIP derivatives (CIPD1, CIPD2, and CIPD3).

The three CIP derivatives, namely CIPD1, CIPD2, and CIPD3, were synthesized using the mixed anhydride method (Figure 7). Specifically, a solution of CIP (10 mmol, 3.858 g) was added to 50 mL of dichloromethane, followed by the addition of triethylamine (20 mmol) while stirring at a temperature below 38 °C to alkalize the solution. Subsequently, ethyl chloroformate (15 mmol) was added until the solution became transparent. Then, 10 mmol of arylamines (m-methoxyaniline, benzylamine, and α-naphthylamine) were added, respectively, and the reaction proceeded for 30 min. The resulting solvent was evaporated under vacuum and the final products were separated and purified through column chromatography using ethyl acetate/petroleum ether as eluent in a ratio of 1:1.

The structures of the three synthesized derivatives were analyzed using proton nuclear magnetic resonance (^1^H NMR) spectroscopy (Advance-600 MHz, Bruker Co., Fällanden, Switzerland) and electron ionization mass spectrometry (Esquire_LC, Bruker Co., Rheinstetten, Germany). The melting points of the three CIP derivatives were determined using a micro-melting point apparatus (X-4, Beijing Tech Instrument Co., Ltd., Beijing, China). The specific parameters are as follows (Supplementary Materials Figures S1 and S2):

CIPD1: 1-cyclopropyl-6-fluoro-1,4-dihydro-4-oxo-7-[(1-(4-carbethoxy)piperazinyl]quinoline-3-[N-(3-methoxyphenyl)]formamide

Molecular formula: C_27_H_29_FN_4_O_5_; Molecular weight: 508.21; Yield: 13%; White powder: m.p. 210–211 °C; ^1^H NMR (600 MHz, CDCl_3_) δ 12.20 (s, 1H, -CONH-), 8.86 (s, 1H, C_2_-H), 8.06 (d, 1H, *J* = 13.0 Hz, C_5_-H), 7.51 (brs, 1H, Ph), 7.32 (d, 1H, *J* = 7.0 Hz, C_8_-H), 7.27 (t, 1H, *J* = 7.5 Hz, Ph), 7.22 (drd, 1H, *J* = 7.9 Hz, Ph), 6.66 (drd, 1H, *J* = 7.1 Hz, Ph), 4.20 (q, 2H, J = 7.1 Hz, -OCH_2_-), 3.83 (s, 3H, -OCH_3_), 3.74−3.68 (m, 4H, piperazine-H), 3.52−3.47 (m, 1H, C-Pr C_1_′-H), 3.28–3.20 (m, 4H, piperazine-H), 1.35 (q, 2H, *J* = 6.6 Hz, C-Pr C_3_′-H), 1.30 (t, 3H, *J* = 7.1 Hz, -CH_3_), 1.20 (q, 2H, *J* = 6.5 Hz, C-Pr C_2_′-H). EI-MS (*m*/*z*): 509.19 [M + H]^+^.

CIPD2: 1-cyclopropyl-6-fluoro-1,4-dihydro-4-oxo-7-[(1-(4-carbethoxy)piperazinyl]quinoline-3-(N-Benzyl)formamid

Molecular formula: C_27_H_29_FN_4_O_4_; Molecular weight: 492.22; Yield: 19%; White powder: m.p. 140–142 °C; ^1^H NMR (600 MHz, CDCl_3_) δ 10.36 (t, 1H, *J* = 5.4 Hz, -CONH-), 8.86 (s, 1H, C_2_-H), 8.04 (d, 1H, *J* = 13.1 Hz, C_5_-H), 7.38 (t, 2H, *J* = 7.5 Hz, Ph), 7.33 (dd, 2H, J = 7.3, 2.7 Hz, Ph), 7.31 (s, 1H, C_8_-H), 7.24 (tt, 1H, *J* = 7.3, 2.7 Hz, Ph), 4.66 (t, 2H, *J* = 5.4 Hz, -CH_2_-Ph), 4.19 (q, 2H, *J* = 7.1 Hz, OCH_2_-), 3.74−3.68 (m, 4H, *J* = 4.3 Hz, piperazine-H), 3.46 (dt, 1H, *J* = 10.8, 3.7 Hz, C-Pr C_1_′-H), 3.28−3.20 (m, 4H, piperazine-H), 1.34 (t, 2H, *J* = 6.6 Hz, C-Pr C_3_′-H), 1.30 (t, 3H, *J* = 7.1 Hz, -CH_3_), 1.17 (d, 2H, *J* = 2.9 Hz, C-Pr C_2_′-H). EI-MS (*m*/*z*): 493.31 [M + H]^+^.

CIPD3: 1-cyclopropyl-6-fluoro-1,4-dihydro-4-oxo-7-[(1-(4-carbethoxy)piperazinyl]quinoline-3-[N-(1-Naphthyl)]formamide

Molecular formula: C_30_H_29_FN_4_O_4_; Molecular weight: 528.22; Yield: 9%; White powder: m.p. 236–238 °C; ^1^H NMR (600 MHz, CDCl_3_) δ 12.84 (s, 1H, -CONH-), 8.97 (s, 1H, C_2_-H), 8.51 (d, 1H, *J* = 7.5 Hz, naph-H), 8.40 (d, 1H, *J* = 8.5 Hz, naph-H), 8.19 (d, 1H, J = 13.0 Hz, C_5_-H), 7.86 (d, 1H, *J* = 8.1 Hz, naph-H), 7.64 (dd, 2H, *J* = 11.7, 7.2 Hz, naph-H), 7.54–7.48 (m, 2H, naph-H), 7.36 (d, 1H, *J* = 7.0 Hz, C_8_-H), 4.20 (q, 2H, *J* = 7.1 Hz, -OCH_2_-), 3.74−3.68 (m, 4H, piperazine-H), 3.52 (dt, 1H, *J* = 10.8, 3.6 Hz, C-Pr C_1_′-H), 3.29−3.22 (m, 4H, piperazine-H), 1.37 (q, 2H, *J* = 6.6 Hz, C-Pr C_3_′-H), 1.31 (t, 3H, *J* = 7.1 Hz, -CH_3_), 1.22 (q, 2H, *J* = 6.5 Hz, C-Pr C_2_′-H). EI-MS (*m*/*z*): 529.22 [M + H]^+^.

### 3.3. Sonodynamic Damage to BSA in the Presence of CIP Derivatives

The experiment was conducted according to the method reported by Chen et al. [28]. Initially, four 50.0 mL brown volumetric flasks were labeled as a-d, and each was filled with 5.0 mL of a 100 μM BSA-Tris-HCl-NaCl solution. CIPD1, CIPD2, and CIPD3 were dissolved in a small volume of DMSO and subsequently diluted with a 0.05 M Tris-HCl-NaCl solution to obtain a stock solution of 100 μM concentration. Then, 10.0 mL of each of the three stock solutions was added to flasks b, c, and d, respectively. All solutions were adjusted to a final volume of 50.0 mL using the Tris-HCl-NaCl solution. Subsequently, 25.0 mL of the mixture solution was transferred into a 50.0 mL conical flask and subjected to ultrasound irradiation for a duration of three hours, while another set of samples consisting of an equal volume (25.0 mL) served as the non-ultrasound-treated control group.

UV–vis spectroscopy was utilized to assess the impact of CIP and its derivatives on BSA by measuring the intensity of the absorption peak, which serves as an indicator for the concentration of aromatic amino acid chromophores present in the protein. Additionally, fluorescence spectra were recorded for both the ultrasound-treated and non-ultrasound-treated groups to assess any damage inflicted on the BSA molecules. The UV–vis absorption spectra were measured using a UV–vis spectrophotometer (UV-2550, Shimadzu Co., Kyoto, Japan) in the range of 200–500 nm. The fluorescence spectra were obtained using a fluorescence spectrophotometer (F-7000, Hitachi Co., Chiyoda-ku, Japan) with an excitation wavelength of 280 nm, excitation and emission slit widths set at 5 nm, and a scan rate of 1200 nm/min. The controllable serial-ultrasonics apparatus (KQ-100, Kunshan Ultrasonic Instruments Co., Ltd., Kunshan, China) was utilized at a frequency of 80 kHz and power of 200 W while maintaining a constant temperature of 37.0 ± 0.2 °C throughout the experiment.

Furthermore, we conducted an investigation into the impact of varying durations of ultrasound irradiation, ranging from 0 to 3.0 h at intervals of 0.5 h, as well as the concentration of derivatives, spanning from 0 to 25 μM with increments of 5 μM, on the damage effect on BSA.

### 3.4. Sonodynamic Antibacterial Activities of CIP Derivatives against E. coli

The antibacterial activities of CIP and its CIP derivatives (CIPD1 and CIPD2) combined with ultrasonic irradiation on *E. coli* were investigated using the plate culture colony counting method. Specifically, a bacterial suspension with a concentration of 1.0 × 10^7^ cfu/mL was mixed separately with solutions of CIP or its derivatives (CIPD1 or CIPD2). The mixtures were then placed in 50 mL conical bottles and exposed to ultrasonic irradiation at an intensity of 1.0 W/cm^2^ for a duration of 30 min, while another sample was made in parallel to serve as a non-ultrasonic control group. Following ultrasonic treatment, 100 μL of bacterial solution was added to the solid medium in each group and evenly spread across the plate. All the plates were incubated at a temperature of 37 °C for a period of 24 h. All procedures were performed in triplicate. The inhibition rates (IR) of CIP and its derivatives against *E. coli* were subsequently determined using colony counting, and were calculated using the following Equation (2):

IR = (N_0_ − N_t_)/N_0_ × 100% (2)

where N_0_ represents the colony counts in the control group and N_t_ represents the colony counts in the CIP and its derivatives group. Additionally, this study investigated the antibacterial effects of different concentrations (2.0 μg/mL, 4.0 μg/mL, and 6.0 μg/mL) of CIP derivatives, as well as various durations (15 min, 30 min, and 45 min) of ultrasonic irradiation.

### 3.5. Investigation of the ROS in Various Solutions with or without Ultrasound Irradiation

#### 3.5.1. ROS Production of CIP and its Three Derivatives

To compare the production of ROS in CIP and its derivatives under ultrasonic irradiation, five 50 mL conical flasks were filled with 10 mL of 25 mM of a diphenylurea hydrazine (DPCI) solution. Flasks 2–5 were then supplemented with 10 mL of CIP and its three derivatives stock solutions, respectively, resulting in a final concentration of 20 μM. The duration of ultrasonic radiation was uniformly set to 2.5 h. Subsequently, diphenylcarbazide oxide (DPCO) in these solutions was extracted using a mixed solution of benzene and CCl_4_ in a ratio of 1:1, and each extract was adjusted to a final volume of 10.0 mL using the extraction reagent. Finally, the UV–vis absorbance of DPCO in these solutions was measured at a wavelength of 563 nm to evaluate their ROS production capability under ultrasonic excitation.

#### 3.5.2. ROS Production of Varying Concentrations of Derivatives and Ultrasonic Irradiation Durations

CIPD1, which has demonstrated significant sonodynamic activity, was chosen for investigating the correlation between ultrasonic irradiation durations and concentrations on ROS generation. To assess the impact of CIPD1 concentration, five 50 mL volumetric flasks were initially filled with 10 mL of a 25 mM DPCI solution. Then, four of the volumetric flasks were supplemented with varying volumes of CIPD1 solutions (100 μM) to achieve final concentrations of 0, 5, 10, 15, and 20 μM, respectively. Each sample solution was divided into two parts: one was transferred into a sealed 50 mL conical flask for ultrasonic irradiation, while the other served as a control group without ultrasonic irradiation. After 2.5 h, DPCO in these solutions was extracted using a benzene–CCl_4_ (1:1) mixed solution and measured at a wavelength of 563 nm following the method described in Section 3.5.1.

The investigation on the impact of ultrasonic irradiation durations involved six conical bottles containing DPCI solution and another six conical bottles containing both DPCI and CIPD1 solutions, with final concentrations of 5 mM for the DPCI solution and 20 μM for the CIPD1 solution. All solutions were subjected to varying durations of ultrasonic radiation: 0, 0.5, 1.0, 1.5, 2.0, and 2.5 h, respectively. Subsequently, the extraction of DPCO was performed using the aforementioned method, with UV–vis absorbance detected at a wavelength of 563 nm.

#### 3.5.3. Identification of ROS Species

The investigation of ROS species generated by CIP derivatives (CIPD1, CIPD2, and CIPD3) and ultrasound irradiation employed the same method. For example, to detect the ROS species produced by CIPD1, 10 mL of DPCI solution (25 mM) and 10 mL of CIPD1 solution (100 μM) were added to each of the five 50 mL volumetric flasks. Subsequently, 10 mL of scavengers-ascorbic acid (Vc), L-histidine (L-His), sodium azide (NaN_3_), and D-mannitol (D-Man) at a concentration of 50 mM were added to four of the flasks, while the remaining flask served as a control group without any quencher added. Finally, all solutions were diluted to scale with the Tris-HCl-NaCl solution. Subsequently, 25 mL of each solution was transferred into five 50 mL conical bottles for sealing and subjected to ultrasonic irradiation. Simultaneously, another portion of 25 mL of the solution was kept as a control group, without undergoing ultrasonic treatment. After 2.5 h, all samples were extracted with a benzene–CCl_4_ (1:1) mixed solution and subsequently diluted to 10.0 mL with the same extraction agent before their UV–vis spectra were measured at 563 nm. The type and quantity of ROS generated by CIP derivatives under ultrasonic conditions can be inferred from the choice of quencher and the disparities in DPCO absorption values with or without ultrasonic treatment.

### 3.6. Statistical Analysis

All data are presented as means ± standard deviation (SD). GraphPad Prism 8.0.1 (GraphPad Software, San Diego, CA, USA, www.graphpad.com, accessed on 15 June 2024.) was used for graph generation and statistical analysis. Significance was evaluated using Student’s T-test and *p* < 0.05 was considered statistically significant.

## 4. Conclusions

In this study, we have developed three novel derivatives of ciprofloxacin (CIPD1, CIPD2, and CIPD3) through structural modifications to the 3-substituents of ciprofloxacin. The synergistic sonodynamic damage effect, combined with ultrasound irradiation on BSA and *E. coli*, was investigated using UV–vis spectroscopy and fluorescence spectroscopy. The results demonstrated strong sonodynamic activities for all three derivatives, with CIPD1 and CIPD2 showing superior efficacy compared to CIP. This could be attributed to the electron-donating substituent’s potential enhancement of the sonodynamic activity of CIP. All three CIP derivatives could be activated to produce ROS following ultrasound irradiation, which may be the potential mechanism underlying their sonodynamic activities. Overall, our findings indicate that the three newly synthesized CIP derivatives, particularly CIPD1 and CIPD2, exhibit significant sonodynamic activity and have promising potential for further development as novel sonosensitizers in non-invasive SDT treatment of various cancers and multi-drug-resistant antimicrobial therapy.

## Data Availability

All data generated or analyzed in this study are included in this published article.

## Supplementary Materials

The following supporting information can be downloaded at: https://www.mdpi.com/article/10.3390/molecules29163735/s1, Figure S1: The ^1^H NMR Spectra of the CIP derivatives; Figure S2: The electron ionization mass spectrometry of the CIP derivatives.
