# Supplementary material for: Investigation into the Sonodynamic Activity of Three Newly Synthesized Derivatives of Ciprofloxacin
Source: Molecules. 2024 Aug 7;29(16):3735. doi: 10.3390/molecules29163735 (PMC11357595; doi:10.3390/molecules29163735)

Supporting Information

Title: Investigation into the sonodynamic activity of three newly synthesized derivatives of Ciprofloxacin

Figure S1. The ^{1}H NMR Spectra of the CIP derivatives

CIPD1：

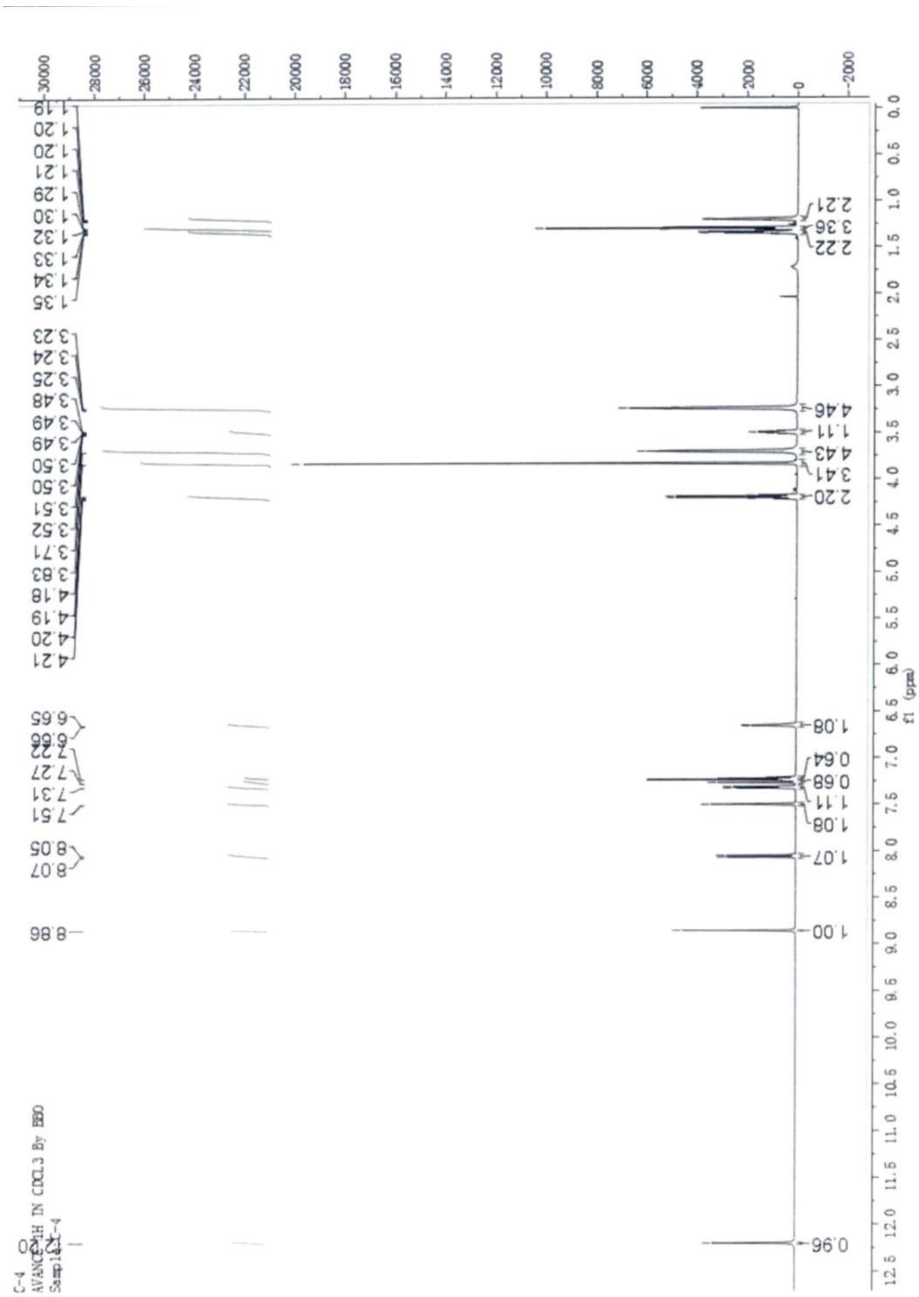

CIPD2：

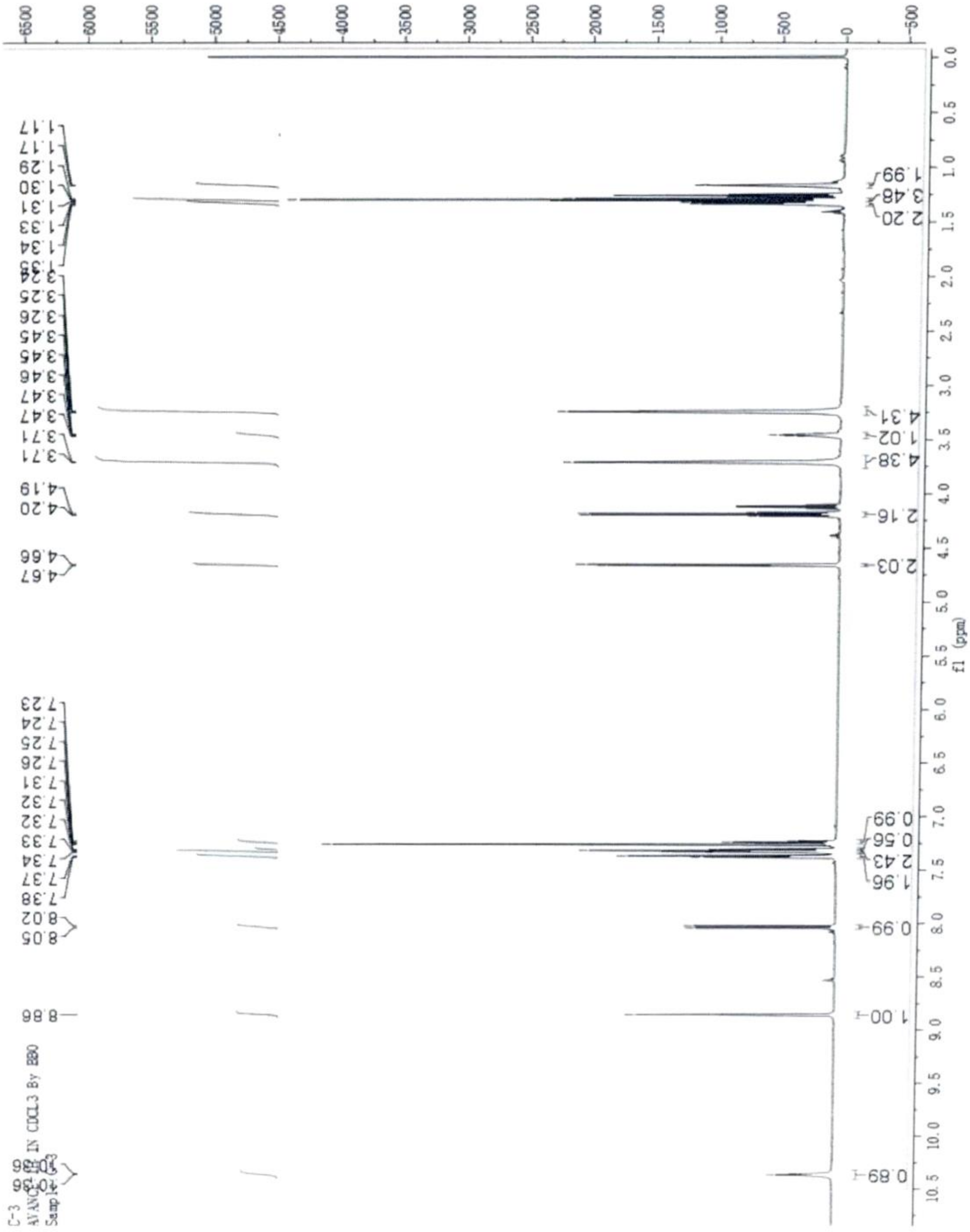

CIPD3：

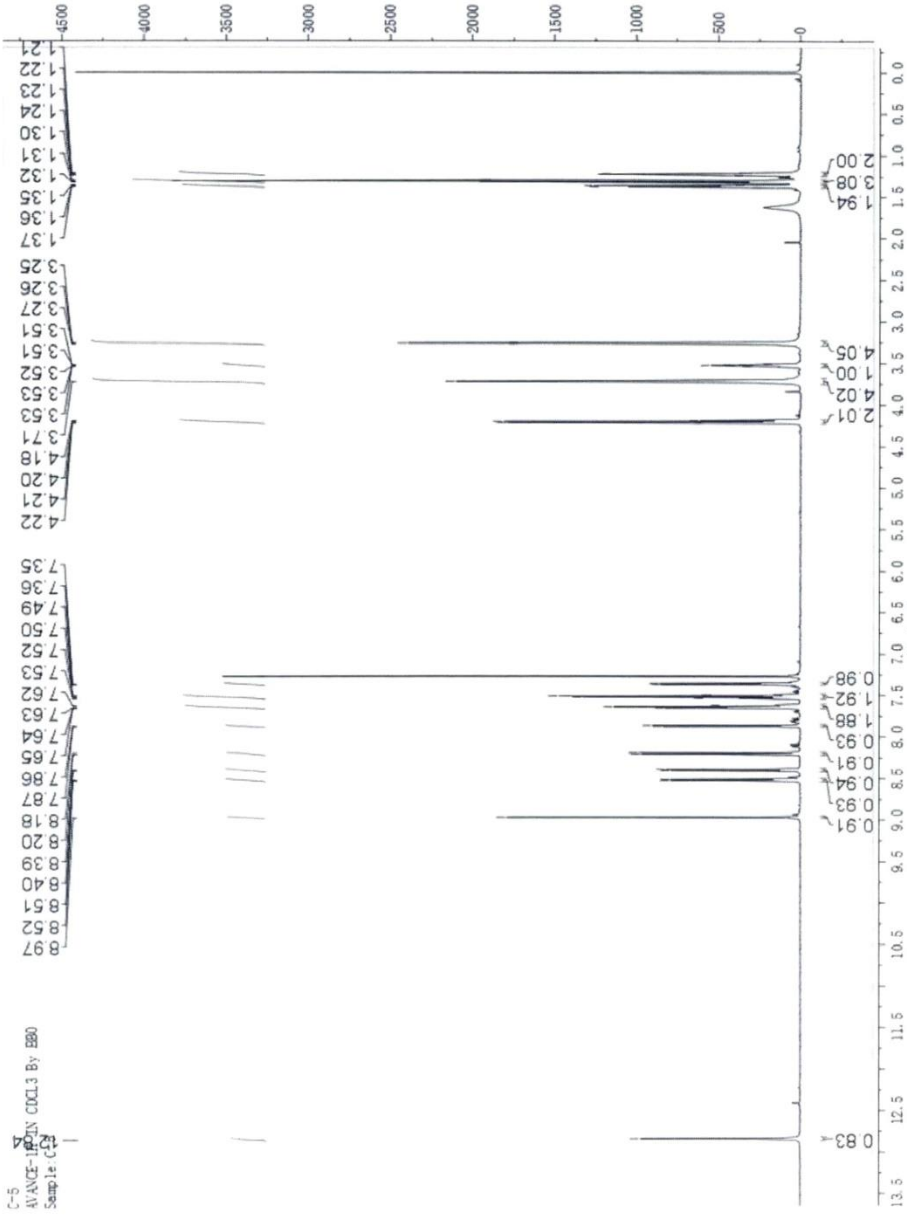

Figure S2. The electron ionization mass spectrometry of the CIP derivatives

CIPD1：

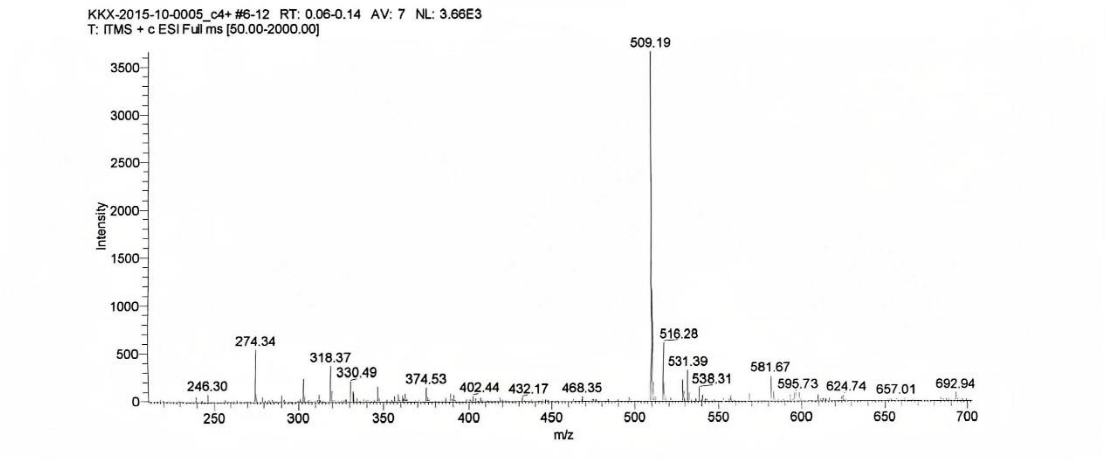

CIPD2：

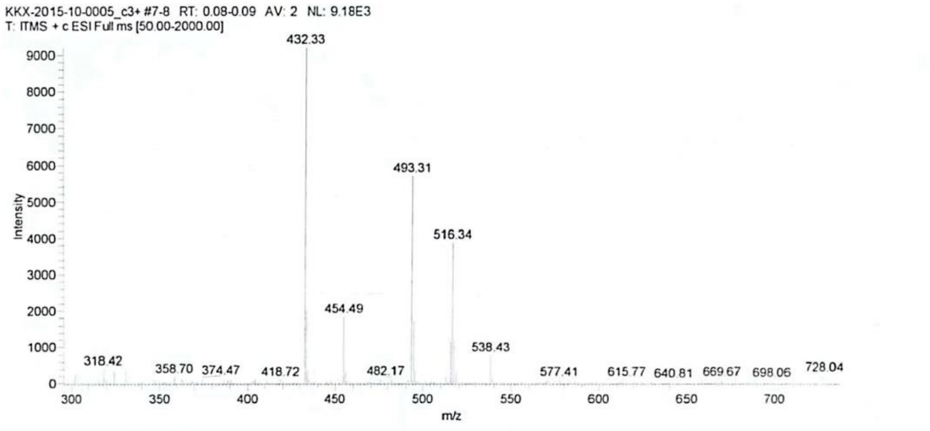

CIPD3:

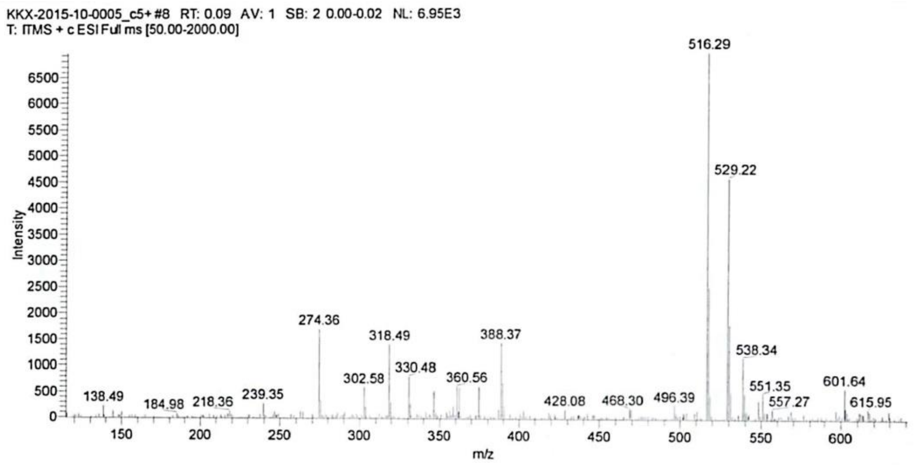

Supplement: Supplementary file 1 [file molecules-29-03735-s001.zip › molecules-3116225-supplementary.pdf]
